# Integrated querying and version control of context-specific biological networks

**DOI:** 10.1093/database/baaa018

**Published:** 2020-04-15

**Authors:** Tyler Cowman, Mustafa Coşkun, Ananth Grama, Mehmet Koyutürk

**Affiliations:** 1 Department of Computer and Data Sciences, Case Western Reserve University, Cleveland, OH 44106, USA; 2 Center for Proteomics and Bioinformatics, Case Western Reserve University, Cleveland, OH 44106, USA; 3 Department of Computer Engineering, Abdullah Gül University, Kayseri 38080, Turkey; 4 Department of Computer Science, Purdue University, West Lafayette, IN 47906, USA

## Abstract

**Motivation:**

Biomolecular data stored in public databases is increasingly specialized to organisms, context/pathology and tissue type, potentially resulting in significant overhead for analyses. These networks are often specializations of generic interaction sets, presenting opportunities for reducing storage and computational cost. Therefore, it is desirable to develop effective compression and storage techniques, along with efficient algorithms and a flexible query interface capable of operating on compressed data structures. Current graph databases offer varying levels of support for network integration. However, these solutions do not provide efficient methods for the storage and querying of versioned networks.

**Results:**

We present VerTIoN, a framework consisting of novel data structures and associated query mechanisms for integrated querying of versioned context-specific biological networks. As a use case for our framework, we study network proximity queries in which the user can select and compose a combination of tissue-specific and generic networks. Using our compressed version tree data structure, in conjunction with state-of-the-art numerical techniques, we demonstrate real-time querying of large network databases.

**Conclusion:**

Our results show that it is possible to support flexible queries defined on heterogeneous networks composed at query time while drastically reducing response time for multiple simultaneous queries. The flexibility offered by VerTIoN in composing integrated network versions opens significant new avenues for the utilization of ever increasing volume of context-specific network data in a broad range of biomedical applications.

**Availability and Implementation:**

VerTIoN is implemented as a C++ library and is available at http://compbio.case.edu/omics/software/vertion and https://github.com/tjcowman/vertion

**Contact:**

tyler.cowman@case.edu

## 1 Introduction

Networks are commonly used abstractions in computational and systems biology [1]. Biological networks include: (i) experimentally identified physical and functional interactions such as protein–protein interactions (PPIs) [31, 34], protein–DNA interactions, synthetic lethality and kinase-substrate associations; (ii) statistically and computationally inferred associations, such as expression quantitative trait loci (eQTL), co-expression networks, regulatory networks and gene-disease associations [3]; (iii) evolutionary relationships based on protein co-evolution, sequence homology, protein and domain families; and (iv) ontologies and functional associations that represent curated knowledge of metabolic and signaling pathways [35], Gene Ontology and phenotype ontologies.

**Biological network databases.** Due to the wide variety of available biological network data, many specialized public databases have been developed to organize and serve data to researchers. Available query interfaces range from retrieval of neighbors of a given vertex (e.g. interacting partners of a given protein) to identification of sub-networks that are enriched in a given set of molecules, or identifying the interactions that are involved in a specific pathway. These queries are often supplemented by a front-end that supports visual exploration of these networks.

**Integration of biological networks.** For more sophisticated queries that involve integration of multiple networks, researchers typically download network data in bulk, often in the form of plain-text edge-lists and process the networks in-house to identify patterns and make computational inferences. This process requires computational expertise and presents several hurdles to the user. For example, the names or identifiers of semantically identical vertices may not be consistent across databases and likely need to be mapped before proceeding. In addition, the integrated network may prohibitively large and contain data that is not relevant to the research question. Thus the input edge-lists are usually filtered based on what the researcher is interested in, e.g. specific tissue, evidence type, a subset of vertices, etc. Once the integrated network(s) are ready for analysis, they are either processed using dedicated algorithms developed by the researcher(s) or loaded into network analysis and visualization software such as Cytoscape [36], a network database like Neo4j [17], or used with thegeneral purpose network libraries such as SNAP [24] or igraph [8].

Many studies implementing such pipelines have repeatedly demonstrated the value of network integration in extracting knowledge from diverse biological data sets. For example, in the context of prioritizing candidate disease genes, known gene-disease associations are integrated with networks of functional and physical association among proteins and clinically informed disease ontologies, enabling a transfer of knowledge between different domains [10, 38]. In the context of cancer, molecular data ranging from mutations and methylation to gene expression and post-translational modifications are integrated with network data to identify driver genes and altered pathways, characterize subtypes [42] and predict drug response [37].

To enable organization and mapping of data from multiple databases, several platforms have been developed. These platforms include UniProt [5], which is protein-centric, and NDeX [33], which focuses specifically on network integration. While these projects enable organization and exploration of networks, they do not provide services for running complex queries on integrated networks.

**Context-specific networks.** Although biomolecular interactions occur in specific biological contexts (e.g. in a specific tissue, under a given set of conditions or as a function of temporal changes), interactions reported in most network databases are generic [13]. To address this limitation of interaction data, many computational methods have been developed to infer tissue-specific networks based on expression of genes across different tissues [30, 40]. Networks that represent statistical associations, such as eQTL are also tissue-specific since the variant may only affect gene expression in a subset of tissues [26]. Similarly, co-expression networks and regulatory networks are context-specific, representing different processes, perturbations or diseases [12]. It has been shown that incorporation of context-specificity enhances the performance of network algorithms in various tasks, including disease gene prioritization [27] and identification of disease-specific regulatory modules [28]. Thus it is essential to take context-specificity into account while integrating biological network data.

**Integration and querying of versioned networks.** Context-specific networks can be abstracted as versions of a generic network, in that these networks are distinct, but related with moderate to large overlaps in their topology (e.g. tissue-specific PPI networks or disease-specific pathways). We call these ‘base versions’. Combinations of these base versions, which we refer to as ‘composite versions’ (e.g. a network that represents a subset of related tissues or a group of clinically similar diseases) enables flexible representation of the relationship between different contexts. Integration of base and/or composite versions of different types of networks (e.g. eQTLs and PPI networks representing a set of tissues) gives rise to ‘versioned heterogeneous networks’. In the current state-of-the-art, when a researcher wishes to use a versioned heterogeneous network, for example, to utilize a different set of tissues and data types or query across versions, the integration process needs to be repeated and a new network data-structure must be created for every version. This is a rather inefficient procedure, both in terms of memory use and compute time, when querying across many integrated network versions.

**Contributions of this study.** Here we develop a framework to enable efficient integrated querying of versioned biological networks. When storing and indexing versioned networks, a desirable property is efficient access to arbitrary versions and a seamless interface for obtaining composite versions (e.g a network representing a set of related tissues). A straightforward method for providing such functionality is to generate and store all relevant combinations of versions. However, the number of possible composites is exponential in the number of explicitly stored versions. Thus pre-computing and storing the desired combinations may not be feasible in most cases.

Motivated by these considerations, we develop VerTIoN, a version-tree-based data structure that enables efficient storage, composition and querying of versioned biological networks. Leveraging significant overlap between different versions of biological networks (e.g. a giant component of the generic human PPI network that represents processes that are common to all human tissues), VerTIoN enables compressed storage, efficient composition and querying of integrated networks. To demonstrate the utility of VerTIoN, we specifically focus on network proximity queries in tissue-specific protein interaction and eQTL networks, since the utility of these algorithms in tissue-specific networks has been well established [27].

To enable flexible processing of proximity queries on versioned networks, VerTIoN offers the following functionalities:

- A novel data structure for efficient storage, composition and access of multiple networks with the capability of use in a multi-user context.
- Composition of networks that represent specified combinations of different versions (networks that represent multiple tissues) and different types of networks (tissue-specific PPI networks, tissue-specific eQTL networks and generic gene–disease associations).
- Capability to process concurrent network proximity queries on these integrated networks in real-time.

We benchmark VerTIoN on tissue-specific collections of networks representing different data types obtained from disparate databases. We also compare VerTIoN’s performance to that of Neo4j, the most commonly utilized graph database solution for storing and querying very large graphs. Our results show that VerTIoN outperforms alternate options in terms of version extraction and composition and consistently processes proximity queries on different combinations of networks in real time.

## 2 Methods

In this section, we first present the sample application of integrating context-specific biological networks. We then describe random walk-based network proximity as an example query on these networks and identify the operations that are required to efficiently compute it for compositions of network versions. Finally, we describe the proposed data structure for efficiently performing these operations.

### 2.1 Integration of versioned biological networks

**Fig. 1:**
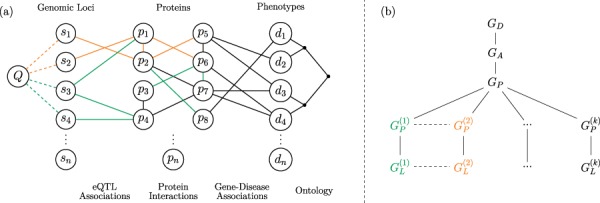
**Versioned heterogeneous network.** (a) An example of a versioned network that is used to model the relationship between genomic loci, proteins and diseases. The vertices consist of genomic loci, proteins and disease phenotypes, edges represent tissue-specific eQTL interactions, tissue-specific PPIs, gene–disease associations and relationships between diseases in the context of a disease ontology. Different versions represent interactions that occur within specific tissues. The interactions that occur in different versions are shown using different colors, where black edges represent generic interactions/associations. A proximity query on this network can be formulated using any subset of loci, proteins, diseases or any combination of these. As an example, vertex *Q* visualizes a sample query that aims to retrieve the diseases most closely associated with one or more genomic loci or a genomic region. (b) The version tree used to represent the integrated heterogeneous network in (a). The root node represents the generic disease ontology network. Version $G_A$ contains the generic gene–disease association network. $G_P$ represents the generic PPI network, consisting of the edges that are shared by the tissue-specific PPIs. The next level of the tree splits off into the different tissue-specific PPI networks, as shown using different colors. At the final level, the corresponding tissue-specific eQTL networks are added.

The framework we propose for integrated querying of tissue-specific networks is shown in Figure 1. Consider a network consisting of disease vertices and edges representing an ontology of clinically defined relationships (i.e. a disease network) and a separate network consisting of proteins and their interactions (i.e. a generic PPI network). As seen in the figure, these two networks can be integrated using a bipartite network of established gene–disease associations [15, 32]. Integration of each tissue-specific PPI network with the generic disease networks results in a different version of the integrated network. Tissue-specific networks are further augmented by adding edges between protein vertices and genomic loci by utilizing eQTL interactions, which are inherently tissue specific.

The objective of the proposed framework is to enable an end-user to construct integrated networks that correspond to any plausible combination (as we define below) of these networks and run complex queries on the resulting integrated network. Formally, the framework illustrated in Figure 1 utilizes the following networks as inputs:

- A ‘generic’ disease ontology network $G_D(V_D, E_D),$ where $V_D$ is a set of diseases and $E_D$ is a set of edges describing the clinical classification of these diseases.
- Tissue-specific PPI networks $G_P^{(i)}=(V_P^{(i)}, E_P^{(i)})$ for $1 \leq i \leq k$, where $V_P^{(i)}$ is a set of proteins that are expressed in tissue $i$, and $E_P^{(i)}$ is the set of interactions between these proteins that occur in tissue $i$. We refer to each of these networks as ‘base versions’.
- A ‘generic’ bipartite gene–disease association network $G_A(V_A, E_A)$, where $V_A= V_P \cup V_D$, $V_P=\cup _{i=1}^{k}{{V_P}^{(i)}}$, $V_D$ is the set of diseases, and $E_A$ is the set of edges denoting association between a gene in $V_P$ and a disease in $V_D$.
- Tissue-specific bipartite eQTL networks $G_L^{(i)}(V_L^{(i)}, E_L^{(i)})$ for $1 \leq i \leq k$, where $V_L^{(i)} = (V_S^{(i)}\cup V_P^{(i)})$, $V_S$ is the set of genomic loci, $V_P$ is the set of genes, and $E_L^{(i)}$ is the set of edges denoting a statistically significant association between the genotype of a genomic locus in $V_S$ and the expression of a gene in $V_P$ in tissue $i$.

We integrate these input networks using a ‘version tree’ as shown in Figure 1b. The version tree first integrates generic networks and subsequently adds different types of networks and tissue-specific versions. The generic PPI network ($G_P$) in this version tree is obtained by taking the intersection of interactions in all tissue-specific PPI networks, i.e. $G_P=(V_P, E_P)$, where $E_P=\cap _{i=1}^{k}{{E_P}^{(i)}}$. The nodes that correspond to tissue-specific versions contain the tissue-specific edges that are not contained in the generic PPI network, i.e. ${\bar{G}_P}^{(i)}=(V_P^{(i)},{\bar{E}_P}^{(i)})$, where ${\bar{E}_P}^{(i)} = E_P^{(i)}\setminus E_P$ for $1 \leq i \leq k$. In Figure 1a, generic edges ($E_D$, $E_A$) are shown in black, tissue-specific edges ($E_P^{(i)}$, $E_L^{(i)}$) are shown in different colors, and PPIs that are common to all tissues ($E_P$) are shown in black. It is possible that a PPI that is not common to all tissue-specific networks may occur in more than one tissue. Such edges are contained in multiple branches of the version tree.

Observe that, in the version tree, there is an edge between any pair of networks that can be integrated through a shared (sub)set of vertices. Using this property, VerTIoN performs network integration using a combination of the following two integration schemes:

- ‘Vertical integration’ is performed by integrating versions alongside a path in the version tree. Vertical integration is always performed by taking a union of the vertices and edges of the networks that are being integrated. This results in permanently stored network versions.
- ‘Horizontal integration’ is performed by integrating two or more sibling networks in the version tree. Horizontal integration can be performed by taking either the union, intersection or some other function of the edges in the corresponding versions (as specified by the user). Horizontal integration can be (and usually is) performed in conjunction with vertical integration. For example, to integrate PPI and eQTL networks representing a set of tissues, VerTIoN first implicitly vertically integrates PPI and eQTL networks for each tissue, then horizontally integrates the resulting PPI+eQTL networks across tissues. Note that vertical integration is generally a query time procedure that generates temporary versions. However, the result of a vertical integration can be saved back to the version tree as a child of any appropriate node for permanent storage.

It is important to note that this version tree structure integrates networks of different types additively, thus representing versions as a sequence of edge additions along a path in the tree. Networks that represent different contexts with the same semantics, on the other hand, are represented by different branches of the tree. This representation enables efficient construction of integrated networks at query time by computing intersections or unions of edge lists. As we discuss below, the data structure implemented in VerTIoN exploits this additive property during vertical integration and is optimized for edge additions. For this reason, although VerTIoN supports vertically integrated deletions as well, it performs additions more efficiently.

### 2.2 Querying integrated heterogeneous networks

We consider a flexible query framework for network proximity queries on integrated tissue-specific networks. A user can formulate their query as follows:

- **Network type:** select the types of networks to be included in the computation from the set $\{G_D, G_P, G_A, G_L\}$, such that the selected networks induce a path in the version tree.
- **Set of tissues:** select a subset $T$ of tissues to be included in the computation.
- **Query type:** select whether the integrated network should contain an interaction if it is present in all tissues in $T$ (intersection query) or in at least one tissue in $T$ (union query).
- **Seed vertices:** select a set of query vertices $Q \subset (V_D \cup V_P \cup V_S)$. The query can be a disease vertex, an internal vertex in the disease ontology that spans multiple diseases, a set of proteins/genes, a set of genomic loci or a genomic region spanning multiple loci or a combination of these.

A query formulated with these selections defines an integrated network $G_C(V_C, E_C)$, which is composed during query time and used to process the query on-the-fly. Here, $V_C$ is a union of the vertex sets of the network types specified by the user. $E_C$, on the other hand is obtained by a series of union and intersection operations. Namely, a union of edges is computed for (i) vertical integration and (ii) horizontal integration when the query type is union. An intersection of edges is computed for horizonal integration when the query type is intersection. Once $G_C$ is constructed, network proximity computation is performed to rank the vertices in the integrated network in terms of their proximity to the seed vertex(s) in the integrated network (as we describe in Section 2.5).

Observe that the number of possible networks that can be composed by a user-defined query is exponential in $k$, thus it may not feasible to compose each of these networks offline and store them. VerTIoN enables online composition of these integrated networks using the version tree described in the previous section and the associated data structure we describe in the next section.

### 2.3 Data structure for storing versioned network topology

In VerTIoN, we develop a novel data structure to store and retrieve the topology of versioned networks. At this level, the data structure is agnostic to any vertex semantics and only refers to vertices using indexes. The proposed data structure is an extension of the compressed sparse row (CSR) data structure, which enables the storage and retrieval of distinct networks [9]. To this end, an instance of the proposed data structure that does not contain multiple versions is nearly identical to a standard CSR matrix.

#### 2.3.1 Standard CSR format

Let $M$ denote the adjacency matrix for undirected network $G(V,E)$. The compressed sparse row matrix format consists of three arrays: (i) $2|E|$-dimensional array *A* contains the non-zero elements of $M$ in row major order. (ii) $2|E|$-dimensional array *JA* contains the indices of the corresponding columns in $M$. (iii) ($V$+1)-dimensional array *IA* stores the starting index for each row of $M$ in the previous two arrays. In other words, if we use $adj_i$ to denote the adjacency list of vertex $i$ in $G$, then

(1) \begin{equation*} adj_i = \textit{JA}[\textit{IA}[i]\;... \; \textit{IA}[i+1] - 1]. \end{equation*}

In other words, the array *IA* stores the starting index in the array *JA* of the list of neighbors for each vertex $i$.

**Fig. 2:**
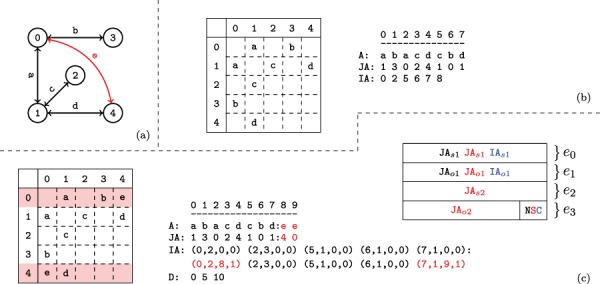
**Versioned compressed sparse row (CSR) format.** (a) A sample two version network in which the first version consists of the black edges and the second version includes an additional edge shown in red. (b) The adjacency matrix and three arrays used to represent the first version of the network. (c) The adjacency matrix and arrays used to store both versions of the network in the proposed data structure and the memory layout for the elements of the augmented $\textit{IA}$ array. For the adjacency matrix, the colons denote where the second version’s data has been appended. For the memory layout, each $\textit{IA}$ element is comprised of four segments $(e_{0..3})$. Note that the type flags $(e_4)$ are stored in the least significant bits of $e_3$. The subscripts $o$ and $s$ refer to a starting index and length respectively in the $A$ and $\textit{JA}$ arrays. The last bits of $e_3$ are used to store a set of flags denoting the row status: normal, split or compressed. These are used to describe how the rest of the entries in $\textit{IA}$ should be interpreted. The meaning of each $(e_{0..3})$ value is color coded based on the flag. Note that only one interpretation is active for each entry of $\textit{IA}$ at a time.

An example network with its adjacency matrix and corresponding CSR representation are shown in Figure 2a and b.

#### 2.3.2 Tracking versions

As shown in Figure 1b, VerTIoN represents different versions of a network as a tree. In this tree, the path from the root to each node represents a different version of the network. Therefore, the version that corresponds to a child vertex in the tree can be obtained by adding edges to the version represented by its parent node. In VerTIoN, the networks along each path in the version tree are represented using a single set of $A$, $\textit{JA}$ and $\textit{IA}$ arrays. For $A$ and $\textit{JA}$, the edges in ancestral versions (i.e. those closer to the root) are reused and only additional edges are appended for the descendant versions. In contrast, the $\textit{IA}$ arrays are replicated for each version such that different versions correspond to non-overlapping segments on $\textit{IA}$. This is illustrated in Figure 2c, which shows the $A$, $\textit{JA}$ and $\textit{IA}$ arrays for the two versions of the network shown Figure 2a. For ease of visualization, the segments of arrays that correspond to different versions are separated by a colon.

VerTIoN tracks versions on a path in the version tree using an $\ell $-dimensional array $D$, where $\ell $ denotes the number of nodes in the path from the root to a leaf of the version tree. For $0\leq d \leq \ell $, the $\textit{IA}$ array of the $d$th version on a path in the version tree is represented as:

(2) \begin{equation*} \textit{IA}^{(d)} = \textit{IA}[D[d] \;... \; D[d+1]-1] \end{equation*}

where $d=0$ for the root version. The $D$ array for storing the two versions of the network in Figure 2a is also shown in Figure 2c.

Observe that the length of the $A$ and $\textit{JA}$ is proportional to the number of edges in a network, while the length of $\textit{IA}$ is proportional to the number of vertices. For this reason, replicating $\textit{IA}$ to effectively compress $A$ and $\textit{JA}$ reduces storage and makes effective use of cache locality. We further compress $\textit{IA}$ using a similar idea, which we discuss later in Section 2.3.5.

To facilitate effective compression of the $A$ and $\textit{JA}$ arrays and efficient retrieval of different versions, we ‘augment’ the IA array with additional features that provide pointers to edge lists of vertices in the parent and child versions.

#### 2.3.3 Augmented IA array

The memory layout for an augmented $\textit{IA}$ element is shown in Figure 2c. As seen in the figure, each element of the augmented IA array is a 4-tuple $\{e_0,e_1,e_2,e_3\}$. For a vertex $i$, the least significant two bits of $\textit{IA}_i[e_3]$ specifies the ‘type’ of $\textit{IA}_i$. The possible values for the type of a row are ‘normal row’ ($N$), ‘split row’ ($S$) and ‘compressed row set’ ($C$).

**Normal row (N).** A normal row corresponds to a vertex with no new edges in the child version. For a normal row, $\textit{IA}_i[e_0]$ represents the starting index of $\textit{JA}$ the adjacency list of the $i$th vertex, while $\textit{IA}_i[e_1]$ represents the number of edges incident to vertex $i$. The $e_2$ and $e_3$ elements are unused. Thus the adjacency list for normal row $i$ is retrieved as:

(3) \begin{equation*} adj_i = \textit{JA}[\textit{IA}_i[e_0]\;... \; \textit{IA}_i[e_0]+\textit{IA}_i[e_1]] \end{equation*}

**Split row (S).** A split row $\textit{IA}_i$ defines two contiguous segments of $\textit{JA}$ that contain the adjacency lists of vertex i. Split rows are used to enable reuse of the edges that already exist in the ancestral versions. $\textit{IA}_i[e_0]$ and $\textit{IA}[e_1]$ represent the starting index and length of the first segment, while $\textit{IA}_i[e_2]$ and $\textit{IA}_i[e_3]$ represent a second segment of values in $\textit{JA}$. Thus the adjacency list for split row $i$ is retrieved as:

(4) \begin{equation*} \begin{array}{l} Segment_1 = \textit{JA}[\textit{IA}_i[e_0]\;... \;\textit{IA}_i[e_0]+\textit{IA}_i[e_1]]\\ Segment_2 = \textit{JA}[\textit{IA}_i[e_2]\;... \; \textit{IA}_i[e_2]+\textit{IA}_i[e_3]]\\ adj_i = Merge(Segment_1,Segment_2).\\ \end{array} \end{equation*}

**Compressed row set (C).** In addition to $\textit{IA}$ elements that represent vertices in the network, additional entries in $\textit{IA}$ are used to compress the $\textit{IA}$ array. When an entry in $\textit{IA}$ is marked as a ‘compressed row set’, it represents a contiguous segment of previously encountered elements in the augmented $\textit{IA}$ array. We explain how $\textit{IA}$ is compressed and reconstructed for compressed row sets in Section 2.3.5.

#### 2.3.4 Adding A child version

When a new network is added to a parent version, the parent version’s $\textit{IA}$ section is copied and appended to the $\textit{IA}$ array. Next, the vertices that have at least one edge added in the new version are updated, appending their values to the $\textit{JA}$ and $A$ arrays and updating the corresponding entry in the $\textit{IA}$ array of the new version. We call these vertices *updated vertices*. For each updated vertex, the update procedure performs one of the following operations: (i) split a normal row, (ii) split a split row or (iii) join a split row.

**Splitting a normal row.** This is the case when a vertex that is represented by a normal row in the parent version gains edges in the child version. Therefore, in the parent version, the $e_0$ and $e_1$ fields of respective $\textit{IA}$ entry point to the adjacency list of the vertex on $\textit{JA}$. The $e_2$ and $e_3$ fields are unused. Thus after appending the new edges to $\textit{JA}$ and $A$, the new range bounds are stored in the fields that are not used by the parent. Consequently, in the child version, the vertex is represented by split row.

**Splitting a split row.** To ensure constant-time access to the adjacency list of a given row regardless of the number of stored versions, VerTIoN maintains that a split row can define at most two separate contiguous ranges. Thus, once a row is split, it cannot be split again. Instead, we merge the second segment of the adjacency list of the vertex ($\textit{JA}[\textit{IA}[e_2], \textit{IA}[e_2]+\textit{IA}[e_3])$) in the parent network with the edges that are added to the child version and append these edges together to the $\textit{JA}$ array. We set the $e_2$ and $e_3$ fields in the $\textit{IA}$ array accordingly. Note that this results in the duplication of edge data for a vertex that gains edges in more than two versions. To reduce the overhead caused by these duplications, we define a third procedure that joins a split row.

**Fig. 3:**
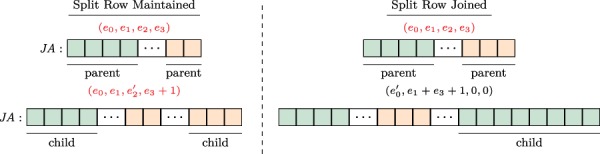
**Joining/maintaining a split row.** The structure of the $\textit{JA}$ array and associated augmented $\textit{IA}$ element, before (upper panel) and after (lower panel) the addition of an edge to the adjacency list of a vertex. In each $\textit{JA}$ array, the green (orange) segment shows the part of the adjacency list that is pointed by the $e_0$ and $e_1$ ($e_2$ and $e_3$) fields of the respective entry in the $\textit{IA}$ array. In the example on the left, the split row is maintained since $(e_1+1)<e_3)$, whereas on the right, the split row is joined since $(e_1+1)>e_3)$.

**Joining a split row.** As discussed above, splitting a split row causes creation of multiple copies of adjacency lists. As a worst case example, consider a chain of $\ell $ versions with repeated addition of $r/\ell $ edges to an initially disconnected vertex. If we repeatedly split rows for this vertex while adding each version, we would use $\Theta (r\ell )$ space to store the edges of a vertex that has $r$ edges in the leaf version. This is asymptotically equivalent to storing each version separately. To alleviate this issue, when new edges are being added to a vertex with a split row, we consider joining its split rows before adding the new edges.

For a vertex, let the number of edges being added to a new version be $t$. We first compare the length of the first segment $(e_1)$ to the length of the prospective new segment $(e_3+t)$. If $e_3+ \geq e_1$, we merge the edges of the vertex in the parent version with its new edges in the child version and append the entire adjacency list as a single segment to the $A$ and $\textit{JA}$ arrays. We then update the $e_0$ and $e_1$ fields of the respective entry in $\textit{IA}$, creating a normal row. When applied to the above worst case scenario, this results in $\lg{\ell }$ copies of $r$ elements and $\lg{\ell }$ copies of $\sum _{j=0}^{\lg{\ell }}\frac{j(j-1)}{2}$ elements resulting in $O(r\lg{\ell })$ space for storing all versions of the adjacency list of such a vertex. Sample cases for maintaining a split row and joining a split row following edge addition are illustrated in Figure 3.

#### 2.3.5 Compressing the IA array

**Fig. 4:**
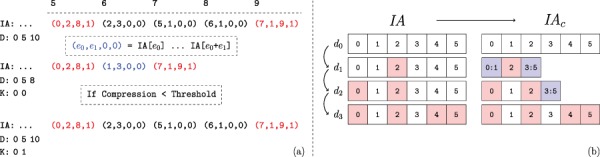
**Compression of augmented IA arrays.** The values of $\textit{IA}$ corresponding to the second network version for the example in Figure 2. (a) The range of unchanged $\textit{IA}$ elements is removed from the array and replaced with a $C$ flag set element representing their range in the previous version. Thus the length of the augmented $\textit{IA}$ array is decreased. However, the compression performance using the first version as a keyframe is determined to be too low. Thus the uncompressed *IA* segment is instead appended, and the key-frame for the new version is set to itself rather than its parent. Note that the initial network version is its own key-frame. (b) Motivating example for key-framing $\textit{IA}$ array segments. The initial version, $d_0$ is not compressed as it does not have a reference version. Versions $d_1$ and $d_2$ retain ranges contained within the initial segment. However, by version $d_3$ the new *IA* segment is unable to be compressed when basing on the initial version.

Recall that, regardless of the extent of the number of edges added, a new $\textit{IA}$ array is created for each child version. Thus, for a branch of the version tree that contains $\ell $ versions, the size of the augmented $\textit{IA}$ array is $\Theta (\ell V)$, where $V$ denotes the set (number) of vertices in the leaf version. If the number of vertices that gain edges in a child version is $\Delta $ such that $\Delta \ll V$, storing an additional copy of $\textit{IA}$ introduces substantial overhead. Motivated by this observation, we use ‘compressed row sets’, which enable an additional level of compression on the $\textit{IA}$ array. This process is illustrated in Figure 4.

While creating a child version, instead of copying the $\textit{IA}$ array of the parent in its entirety, we use entries in $\textit{IA}$ to point to contiguous segments on the $\textit{IA}$ array of an ancestor (as directed by the parent). To distinguish compressed row sets from other types of entries (normal row and split row, both of which point to the adjacency lists of a vertex in the $\textit{JA}$ array) in the $\textit{IA}$ array, we use the least significant two bits of the $e_3$ field. With this representation, the $\textit{IA}$ array of a version needs to to be decompressed before the version can be accessed. Letting $\textit{IA}^{(d)}$ denote the decompressed $\textit{IA}$ array for the $d$th version on a path from the root of version tree, the compressed $\textit{IA}$ array is decompressed as:

(5) \begin{equation*} IA^{(d)} = \underset{D[d]\leq i < D[d+1]}{\operatorname{{\texttt{Concatenate}}}}\{\textit{IA}[\textit{IA}[i].e_0\;... \; IA[i].e_0+IA[i].e_1]\}. \end{equation*}

Here, the least significant two bits of $\textit{IA}[i].e_3$ are set to $C$ for $D[d]\leq i < D[d+1]$. Observe that, given the (compressed) $\textit{IA}$ array of the parent, the compressed $\textit{IA}$ array for a child version can be computed in $O(V)$ time without explicitly constructing the decompressed $\textit{IA}$ array. The decompression of the $\textit{IA}$ array is also performed in $O(V)$ time, which is asymptotically equivalent to the time required to make a copy of the decompressed $\textit{IA}$ array.

The compression procedure maintains that (i) all compressed indexes that are common among multiple versions refer to the version that is closest to the root of the version tree and (ii) a version segment that is referenced by another version cannot contain any compressed elements. This ensures that decompressing an *IA* index does not require traversing across multiple versions. The root version’s augmented $\textit{IA}$ segment trivially fulfills this requirement.

#### 2.3.6 Key-framing the IA array

Consider a chain of network versions in which most of the indexes in last version’s *IA* segment have changed at least once. Based on the previously asserted constraints, *IA* compression is based on the initial version. However, as most indexes have changed, there are few repeated ranges between the two versions. Thus compression of the last *IA* array segment is not feasible. We use *IA* key-framing to address this issue. The key-framing procedure is illustrated with an example in Figure 4b. We define key-frame versions as those that satisfy the constraint that no compressed elements exist within their *IA* array segment. Thus the initial network version also represents an *IA* key-frame. We introduce the array $K$ to store each network version’s key-frame version. After appending a new version, the compression ratio that is obtained by the new *IA* segment when compressing based on its parent’s key-frame version is checked. If the ratio between the compressed and uncompressed *IA* segment is lower than a threshold $h$, then the procedure works as previously described and the new version’s key-frame version is set to its parent’s. However, if the ratio is greater than $h$, then the uncompressed *IA* segment is appended and the new version’s key-frame is set to itself. Note that a network version’s parent does not need to be the same as its $\textit{IA}$ key-frame and often is not.

### 2.4 Version extraction and composition

Data locality is important for many algorithms on graphs, including network proximity queries that require repeated matrix-array multiplications. In the general, for a set of versioned networks, locality is optimal when they are all stored separately. However, since a set of versions has an exponential number of possible compositions, it is not feasible to pre-compute and store all of them. Therefore, VerTIoN is designed to enable efficient optional extraction (vertical integration) and composition (horizontal integration) of any combination of versioned networks at query time.

**Vertical integration.** Here, the integrated network that is queried corresponds to a single node in the version tree. These versions are permanently stored within the tree structure and the following extraction procedure is not required to query them. However, depending on the network size and the query performed it may, though not necessarily, be more efficient to do so in order to improve data locality. For example, obtaining a vertex’s neighbors would not benefit from extraction. Version extraction is performed by iterating over the edge and vertex data relevant to the queried version and generating a network in the form of a standard CSR representation. Thus any network algorithm designed for use with a CSR network is compatible with VerTIoN. Note that this procedure also removes parts of the network that are not reachable from the query vertices. The time required to extract a version via vertical integration is linear in the number of edges in the extracted network.

**Horizontal integration.** Since the networks that are on separate branches of the version tree represent the same semantics, two or more of these networks can be integrated horizontally to compose versions that represent multiple contexts. In the example we study here, horizontal integration can be performed as the union or intersection of different tissue-specific PPI and eQTL networks. For example, the user can query a network composed of interactions that occur in the liver ‘and’ pancreas or a network composed of interactions that occur in the liver ‘or’ pancreas. While performing horizontal integration, VerTIoN takes advantage of the high degree of overlap between the networks on different branches of the version tree. In order to horizontally integrate networks on $b$ branches of the version tree (i.e. compose a network that represents $b$ tissues), VerTIoN first makes a pass over the $\textit{IA}$ array to partition the set of vertices into two disjoint sets, $S_p$ and $S_n$. The set $S_p$ contains the set of vertices whose $\textit{IA}$ arrays are identical over the $b$ branches and $S_n=V \setminus S_p$. Thus, when there is a large degree of overlap in the tissue-specific networks, we have $|S_p| \gg |S_n|$. For all vertices in $S_p$, the edges in the composed version are gathered by a single pass over the $\textit{JA}$ and $A$ arrays. For the vertices in $S_n$, the intersection and/or union of edges across versions can be computed by making $b$ passes over the $\textit{JA}$ and $A$ arrays. Thus horizontal integration of $b$ branches requires $\mathcal{O}(bE)$ time.

**Fig. 5:**
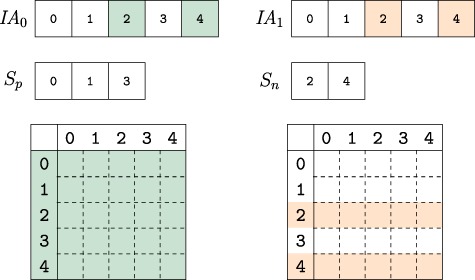
**Composing versions.** The uncompressed *IA* array segments for the versions composed are compared. Colored indexes represent elements that are changed from their parent version. The vertex indexes are partitioned into two sets based on whether they are identical across all composed versions $S_p$ or not $S_n$. When collecting the edge weights from each version, the indexes in $S_p$ only need to be accessed from one version while those in set $S_n$ must be accessed for each version, thus reducing the number of memory accesses to extract and compose network versions.

### 2.5 Proximity and diffusion queries on integrated networks

A useful set of tools that has been central to many network analysis algorithms takes the form of network diffusion algorithms and proximity queries [7]. They commonly utilized to diffuse molecular data across a network to obtain a smooth representation of molecular activity profiles [16, 37]. Similarly, network proximity queries are frequently used to rank/prioritize a set of ‘candidate’ biological entities (e.g. genes in a certain genomic region) in terms of their functional association with a given ‘query’ (or ‘seed’) entity (or a set of query entities, e.g. a disease of interest or a set of proteins that are differentially phosphorylated in a certain condition) [11, 20, 38].

Owing to the common use of proximity and diffusion algorithms, there have been efforts in improving the efficiency of their computation. These efforts are motivated by multiple factors: (i) repeated computation is often needed to assess statistical significance [29] and (ii) querying of networks in an online multi-user context as opposed to bulk data downloads followed by in-house computational analyses. However, these improvements have not yet translated into efficient querying of versioned networks. For a network proximity query service, utilizing a versioned query structure over a full database solution can drastically reduce the amount of space required to store tissue-specific network versions and vastly increase the query throughput and reduce resource utilization. Motivated by these considerations, we assess the performance of VerTIoN in the context of processing proximity queries on integrated tissue-specific networks. The framework we consider here enables a highly flexible query interface by efficiently tackling two key challenges: (i) the user can choose any composition of network versions (e.g. any subset of tissues) and (ii) the queries are processed in a multi-user context.

A proximity query for set of vertices $Q$ on a given network $G_C(V_C, E_C)$ seeks the computation of a array $x_Q$ where $x_Q(v)$ quantifies the proximity of vertex $v \in V_C$ to $Q$ in $G_C$. Network proximity can be quantified using a variety of methods, including random walk with restarts [18, 41] and network propagation [39], among others. A majority of these network proximity measures require the solution to a linear system that involves iterative matrix-array multiplications. As a representative for this type of computation, we here focus on random walk with restarts (RWR)-based proximity queries. It should be noted that the proposed framework either directly applies or can be extended to other types of network proximity measures as well.

RWR-based proximity to a given set of query verticess $Q$ on network $G_C$ is defined as

(6) \begin{equation*} x_Q = (1-\alpha)W_C x_Q+\alpha r_Q, \end{equation*}

where $W_C$ is the stochastic matrix derived from the adjacency matrix of $G_C$ by normalizing all columns by their column sum, $r_Q$ is a $|V_C| \times 1$ array withe $r_Q (v)=1$ if $v\in Q$ and $0$ otherwise, and $\alpha $ is the damping factor (or restart probability) that is used to tune the locality of the proximity measure (a larger $\alpha $ corresponding to a more localized search).

Standard computation of $x_Q$ involves power iterations, where $x_Q^{(0)}$ is set to $r_Q$ and $x_Q^{(t+1)}$ is computed using $x_Q^{(t)}$ on the right-hand side of Equation 6, until $||x^{(t+1)}_Q - x^{(t)}_Q|| < \epsilon $, where $\epsilon $ is a pre-defined stopping criterion to indicate convergence. The rate of convergence of this computation is determined by $\alpha $, i.e. it can be shown that $||x^{(t)}_Q - x^{(*)}_Q|| <(1-\alpha )^t ||x^{(0)}_Q - x^{(*)}_Q||$, where $x^{(*)}_Q$ denotes the desired solution. The number of iterations for this standard iterative computation can be quite large for very large networks, prohibiting real-time processing of proximity queries on networks that are of practical interest.

As we have shown recently [6], the computation of RWR-based proximity can be drastically accelerated using Chebyshev polynomials, where the iterates in the computation are revised as:

(7) \begin{equation*} y_Q^{(t+1)} = \frac{2\zeta}{(1-\alpha)\zeta_{t+1}} \left( Wy_Q^{(t)} + \alpha r_Q \right) - \frac{\zeta_t - 1}{\zeta_t + 1}y_Q^{(t-1)}, \end{equation*}

where $y_Q^{(0)}=0$, $y_Q^{(1)}=r_Q$ and $\zeta _t$ denotes a series derived from Chebysev polynomials. It can be shown that this iterate converges to $x^{(*)}_Q$ faster than the standard power iteration, i.e. $||y^{(t)}_Q - x^{(*)}_Q|| <2\mu ^t ||y^{(0)}_Q - x^{(*)}_Q||$, where

(8) \begin{equation*} \mu=\frac{2(1-\alpha)}{2+\sqrt{2\alpha-\alpha^2}} < 1-\alpha. \end{equation*}

Thus the iterate $y$ converges much faster than the iterate $x$ since an exponential function of $\mu $ decays at an exponentially faster rate than an exponential function of $1-\alpha $. We utilize these optimizations in our implementation of VerTIoN.

## 3 Results

In this section, we first present our experimental set-up by describing the data sets we use, how tissue-specific networks are represented as different versions and the metrics used to evaluate algorithm performance. We then present our results on the storage requirements for different approaches to handling the outlined versioned networks. Finally, we compare the runtime of network proximity queries for VerTIoN and Neo4j and comprehensively characterize the effect of different types of queries and compositions of versions on query processing time.

### 3.1 Experimental setup

#### 3.1.1 Data sources

The inter-phenotype connections and vertices, obtained from the Human Phenotype Ontology, are rooted at a single vertex with deeper vertices representing more specific disease phenotypes [21] *(releases/2018-07-25)*. We connect these phenotype vertices to the protein vertices using associations obtained from DisGeNET [32] *(curated_gene_disease_associations 2018-07-20)*, using Unified Medical Language entries [4] to match appropriate phenotype names between the two databases. The tissue-specific protein interaction sub-networks are obtained from the Integrated Interactions Database [22] *(iid.human 2018-05)* after filtering out the predicted interactions. Tissue-specific eQTL interactions are obtained from the Genotype-Tissue Expression Project [26] *(GTEx_Analysis_v7_eQTL)*. For each eQTL, a vertex is added to the network representing its locus and an edge is added between that vertex and the protein vertex for the gene it acts on.

#### 3.1.2 Network versions

We construct the network version tree shown in Figure 1, consisting of 41 different biological networks, using vertical integration. The initial version consists of only the disease ontology connections $(G_D)$. We create the second version by adding the associations between disease vertices and proteins $(G_A)$. The next 20 versions are constructed by adding 19 tissue-specific PPI networks and the generic PPI $(G_P)$. The last 19 versions add the tissue-specific eQTL associations $(G_L)$ to their respective protein versions. The number of vertices and edges in each version is shown in Table 1.

**Table 1:** **Scale of the networks.** For the tissue-specific PPI and tissue-specific PPI + eQTL versions, the number of vertices and edges are shown. The number of edges for the union of the tissue-specific networks is also shown to highlight the extent of the overlap between the tissue-specific networks

Tissue	PPI: $G^{(1...19)}_P$		PPI + eQTL: $G^{(1...19)}_{L}$	
	#Vertexes	#Edges	#Vertexes	#Edges
Disease ontology	10 843	14 361	-	-
Disease association	15 987	28 706	-	-
Adipose	24 164	294 914	36 285	307 126
Adrenal	24 036	286 741	35 975	298 777
Amygdala	24 200	285 681	36 293	297 899
Brain	24 332	291 391	36 593	303 766
Heart	24 203	293 691	35 891	305 450
Liver	23 851	282 631	35 422	294 276
Lung	23 937	287 438	36 016	299 588
Mammary	24 182	295 060	36 447	307 405
Ovary	24 161	296 297	36 235	308 477
Panaceas	24 087	286 588	35 832	298 408
Pituitary	24 587	300 355	37 244	313 106
Prostate	23 989	288 826	36 106	301 090
Salivary	24 242	293 127	36 460	305 437
Skeletal muscle	23 982	284 589	35 424	296 102
Small intestine	24 428	307 137	36 928	319 753
Spleen	23 809	285 660	35 644	297 582
Stomach	24 323	305 383	36 684	317 824
Testes	24 481	300 701	37 391	313 684
Uterus	24 098	288 368	36 080	300 455
All-union	26 396	356 747	268 285

#### 3.1.3 Performance metrics

We assess VerTIoN’s performance systematically from three different perspectives: comparison of storage effectiveness against state-of-the-art network database Neo4j, comparison of query processing time against Neo4j and processing time on composed tissue-specific networks (i.e. for queries that involve selection of multiple versions by the user). Since both VerTIoN and Neo4j are intended for multi-user applications, we compare the query processing time of VerTIoN against Neo4j in the context of a server application hosting multiple tissue-specific PPI networks serving parallel requests for RWR queries from different clients. In these experiments, each query is based on a randomly chosen version and a randomly chosen query vertex. To assess the performance of VerTIoN in a multi-user context, we implement a simple TCP socket server hosting the versioned PPI network, accepting RWR queries in which the user can select a version and vertex. To reduce noise from network effects, we run the server and client applications on the same machine for both Neo4j and VerTIoN. Using this set-up, we assess the query processing time of VerTIoN and Neo4j as a function of the number of parallel queries that are submitted to the server. All tests are performed on a 64 CPU-E5-4620 @ 2.20 GHz server with 500 GB of memory.

#### 3.1.4 Versioned Neo4j structure

We find that as Neo4j is not structured to be used efficiently in a multi-network context, supporting this feature is not straightforward. Indeed, filling this important gap in network database applications is a major motivation for this study. Nevertheless, for the purpose of comparing VerTIoN against the existing infrastructure provided by Neo4j, we consider two main approaches for implementing multi-network querying: (i) storing each network version as a separate connected component within the database and using vertex properties specifying the version, or (ii) storing all versions as a union network and adding properties to every vertex and edge detailing which versions they exist in. The former method trades space usage for version access speed, while the latter saves space but incurs a $O(D)$ complexity operation to accessing data from a version. Since the access time for a network using VerTIoN does not depend on the number of versions stored, we use the former implementation of versioned networks in Neo4j for comparison, as this provides a more fair comparison in favor of Neo4j.

### 3.2 Compression

**Fig. 6:**
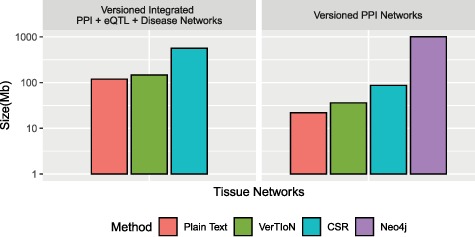
**Compression performance.** Comparison of different methods in terms of the storage space they require for the tissue-specific PPI versions alone and the full set of PPI+eQTL+disease networks outlined in Figure 1. Here CSR refers to the total space required for storing each version separately with a standard CSR representation, whereas VerTIoN refers to the total space required by VerTIoN to store all versions, by exploiting the edge overlap.

We evaluate the storage efficiency of VerTIoN by comparing the space required to store all 41 network versions using the versioned structure as well as independently with standard CSR matrices. We do not consider composite versions while assessing storage effectiveness, since storing all combinations of horizontally integrated versions is clearly not a feasible option. These results are shown in Figure 6. We find that VerTIoN achieves a compression ratio of almost $4x$ compared to storing individual CSR matrices. We also compare to the storage used by representing all versions as plain-text vertex and edge lists. While storing all versions in plain text requires about half the storage required by VerTIoN, plain text is not suited well to performing network queries and represents an extreme of the storage-runtime trade off.

Due to the lack of any compression techniques between versions using the separate connected components approach on Neo4j, and its database overhead, we find that VerTIoN requires roughly $2\%$ of the space of Neo4j to store the tissue specific PPI networks alone.

### 3.3 Query throughput

**Fig. 7:**
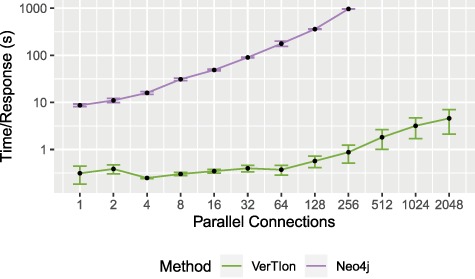
**Throughput for random walk-based network proximity queries.** The average time until server response for a set of parallel connections using a Neo4j database installation and VerTIoN. After 256 parallel requests, Neo4j becomes unstable and begins failing to respond to queries.

We evaluate the runtime performance of VerTIoN as compared to Neo4j in a multi-user context. For both methods we utilize a local server providing access to the tissue-specific PPI networks. We vary the number of parallel user connections/queries, measuring the average time a client waits for a response. The query vertex and tissue network version is randomized for each RWR query, using $\epsilon = 10^{-12}$ and $\alpha = 0.05$. For each number of connections considered, we report the average and standard deviation of query response time for that number of connections. The result of the throughput analysis is shown in Figure 7. Query response time for a single client is shown as a function of the number of parallel queries that are submitted to the server.

As seen in the figure, VerTIoN completes all queries successfully for up to 2048 parallel queries tested. VerTIoN’s query response time is also highly robust to load, with response time remaining around a few seconds even for 2000 queries. In contrast, the response time for Neo4j increases rapidly. Furthermore, Neo4j starts dropping queries as the number of parallel requests exceeds 256. The vast performance difference perfectly illustrates the trade-off between a generalized and a specialized approach. However, note that while these approaches are orthogonal they are not necessarily mutually exclusive. Data-structures such as VerTIoN can be used in conjunction with graph databases as a query acceleration layer. Ultimately, these results clearly show that the data structure and querying framework implemented in VerTIoN is highly promising in making real-time processing of multi-user queries on versioned networks possible.

### 3.4 Composing versions based on tissue combinations

Network composition queries can indeed be very useful and relevant in many biological applications as a pipeline operation for other queries. For example, a researcher may be interested in evaluating genomic loci for their protein proximity in tissues known to be relevant to a specific disease or phenotype such as type II diabetes.

**Fig. 8:**
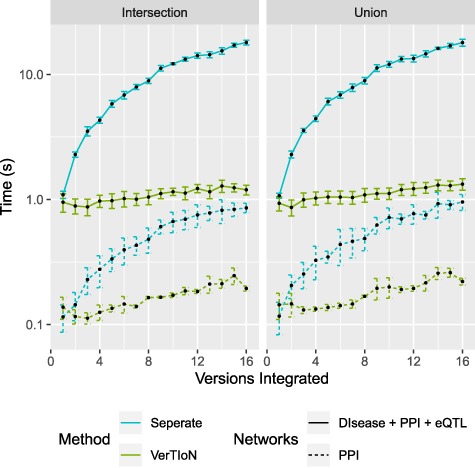
**Processing time for integrating versions.** Shows a run-time comparison between loading and integrating tissue-specific networks when stored separately as CSR matrices and the VerTIoN structure. Each point represents 10 iterations of randomly chosen sets of network versions.

One of the advantages of VerTIoN is its ability to enhance the real-time composition and querying of arbitrary combinations of versions (horizontal integration). We compute the composition time for varying combinations of the tissue-specific networks. Here we consider integration through both edge union and intersection operations. In Figure 8 extraction time is plotted as a function of the number of networks in the composite.

We find that when integrating many network versions, the run-time is dominated by data access either from disk or memory. This is also true even when VerTIoN specific processing is taken into account (sorting and merging split rows). The result of this is that VerTIoN is exceptionally effective for arbitrary compositions as it efficiently loads all of the compressed versions at once, regardless of how many are integrated. This is in contrast to when the versions are stored separately where more data with worse localization needs processing. This large overhead can be seen by the drastic increase in run-time for separate storage as the number of integrated versions increases.

## 4 Conclusion

Here we present VerTIoN, a data structure for efficiently storing, integrating and querying versioned biological network data. By applying a version tree to the compressed sparse row data structure, we enable real-time queries on arbitrary compositions of heterogeneous biological network data. While in this paper we have focused primarily the additive version tree model for tissue-specific biological networks, it is important to note that the presented method is far more general and is not limited to an additive model.

VerTIoN can be used with many other types of context-specific networks. For example, functional association among phosphorylation sites, where each version includes sites that are identified in a different study [2, 25]. Similarly, signaling pathways that are specific to different cancers can be considered as different versions [19] and metabolic networks of different organisms can be organized as different versions [14, 23].

We find that for applications focusing on random walk-based proximity computation, our method provides an exceptionally efficient query structure both in terms of memory use and run-time. It is more accurate to think of VerTIoN as a highly organized way to represent versioned network data rather than a database itself. By providing a compressed structure for storing discrete networks versions, it also enables the ability to quickly compose those versions on the fly. This allows for far more complex queries on arbitrary combinations of stored versions. Ultimately, VerTIoN is not meant as a competitor to databases such as Neo4j, it is an orthogonal but complementary approach to querying versioned network data. We posit that a VerTIoN-like structure could even be used as a query acceleration middle layer for more traditional network databases.
